# Influence of Tumor Stroma on the Aggressiveness of Poorly Cohesive Gastric Carcinoma

**DOI:** 10.3390/jpm14020194

**Published:** 2024-02-09

**Authors:** Giorgio Malpeli, Federica Filippini, Fabrizio Tedone, Lorena Torroni, Mariella Alloggio, Claudia Castelli, Mariagiulia Dal Cero, Roberto Perris, Anna Tomezzoli, Giovanni De Manzoni, Maria Bencivenga

**Affiliations:** 1 Department of Human Sciences for the Promotion of the Quality of Life, San Raffaele Roma Open University, 00166 Roma, Italy; giorgio.malpeli@uniroma5.it; 2 Unit of General and Upper GI Surgery, University of Verona, 37126 Verona, Italy; fabrizio.tedone@univr.it (F.T.); maria.bencivenga@univr.it (M.B.); 3 Unit of Epidemiology and Medical Statistics, Department of Diagnostics and Public Health, University of Verona, 37126 Verona, Italy; lorena.torroni@univr.it; 4 Department of Pathology, Verona University Hospital, 37126 Verona, Italyanna.tomezzoli@aovr.veneto.it (A.T.); 5 Section of Gastrointestinal Surgery, Hospital del Mar Medical Research Institute (IMIM), Universitat Autònoma de Barcelona, 08003 Barcelona, Spain; 6 Centre for Molecular and Translational Oncology (COMT), University of Parma, 43121 Parma, Italy; roberto.perris@unipr.it

**Keywords:** gastric cancer, intratumoral stroma, poorly cohesive, diffuse type

## Abstract

Tumor-stroma crosstalk promotes the adaptation of cancer cells to the local microenvironment and sustains their growth. We assessed the quantitative and qualitative impact of intralesional stroma on clinic-pathological features and the prognosis of poorly cohesive gastric cancer (PCGC) variants. Tissue microarrays including 75 PCGC specimens were immunostained for cytokeratin 8/18 and α-smooth muscle actin to assess the relative proportion of neoplastic cells versus stromal components and the cases were subsequently divided into stroma-rich (SR) and stroma-poor (SP) tumors. Stromal status is significantly associated with the depth of tumor invasion. Patient survival rate was found to be higher in the SP compared to the SR tumor group and, hence, abundant stroma was identified as a significant risk factor in univariable analysis but had no independent prognostic impact. We also investigated the mRNA levels of KRT8 and the associated transcriptional signatures using the molecular data of 82 PCGC cases divided into KRT8-high and KRT8-low groups. KRT8-high tumors were enriched in proteins localized in the extracellular compartment and their expression levels correlated with longer survival in the KRT8-high group and shorter overall survival in the KRT8-low group. Comprehensively, we find that relative intralesional stromal content is a marker of aggressiveness in PCGC tumors and that extracellular proteins characterize functionally and clinically different PCGC subgroups.

## 1. Introduction

Gastric cancer (GC) is the fifth most frequent cancer and the third leading cause of cancer-related death worldwide [1]. Although diagnosis and treatment strategies have been improved in the past decades, overall survival rates remain poor. Furthermore, in recent times, the epidemiology of GC has changed [2,3] as evidenced by an increasing incidence of the diffuse subtype (i.e., poorly pohesive histotype according to the WHO classification [4]) known to display a more aggressive behaviour. There is therefore an urgent need to go more in depth into the biology of poorly cohesive tumours in order to disclose specific and potentially targetable molecular pathways associated with neoplastic progression. Particular attention may in this context be paid to the unfolding of molecular interactions between cancer cells and tumor microenvironments predicted to promote tumour invasiveness, chemoresistance, and metastatic spread [5,6,7].

Intralesional stroma is composed by an acellular part, the extracellular matrix and associated components, and a cellular part consisting of fibroblast, neovessels, and inflammatory cells (such as infiltrating lymphocytes, neutrophils, macrophages, and other cells of the myeloid linage). The major component of the extracellular matrix is collagen, while the cellular part is characterized by the presence of myofibroblasts, distinguishable by the high levels of α-smooth muscle actin (α-SMA) expression and joined by a specific subset of fibroblasts known as “cancer-associated fibroblasts” (CAFs). CAFs are believed to play a central role in the interrelationship between neoplastic cells and the surrounding stroma and exhibit an essential function in gastric cancer progression [8,9]. Quantitative and/or qualitative variations in the stromal component, and/or modulations in the interaction between stroma and neoplastic cells might have a prognostic impact and could represent a potential target for new drugs [10]. Accordingly, recent studies have investigated the tumor-stroma ratio in several cancer types and have suggested that high stromal content may serve as an independent indicator of poor prognosis in solid tumors of the oesophagus, breast, colon, nasopharyngeal apparatus, and liver [11,12,13,14,15]. These data have independently been confirmed by two meta-analyses, one of them focusing on cancers of the digestive tract [16,17]. Nevertheless, in gastric tumors the clinical significance of the tumor-stroma ratio is still undefined. Here, we focused on poorly cohesive carcinomas, both because of their recent increase in incidence and due to their peculiar histopathological features, which warrant the classification of these tumors as a distinct histotype of gastric cancer. The prevalent histopathological trait of this variant remains the individual arrangement of the neoplastic cells, which may occasionally form smaller aggregates embedded in a variable amount of stroma. In this study, we have addressed the impact of a stromal compartment on clinical-pathological features and the prognosis of patients presenting this gastric carcinoma entity.

## 2. Materials and Methods

### 2.1. Samples

We retrospectively reviewed the clinical and pathological data of 106 patients diagnosed with PCGC who had undergone a gastrectomy during the years 2005 to 2014 at the General and Upper GI Surgery Division of the University of Verona (Italy). Patients were excluded from the study if they had presented a neoplastic lesion smaller than 1 cm because of the restriction of precise evaluation of the epithelial versus stromal proportion in such a small tissue specimen. Following the application of the selection criteria, specimens from 75 PCGC patients were included in the study.

Gene expression analyses were based on 82 GCPC samples, divided into 69 diffuse GC and 13 signet ring cell cases and constituting a subset of a larger cohort including 415 samples for which molecular data is part of the stomach adenocarcinoma, Firehose Legacy study, TCGA (cBioPortal at https://www.cbioportal.org (accessed on 28 December 2023) and 240 samples from patients diagnosed with diffuse GC and representing a subset of 875 patients reported by KM plotter at https://kmplot.com/analysis/index.php?p=service&cancer=gastric (accessed on 28 December 2023)).

### 2.2. Immunohistochemistry

**Figure 1:**
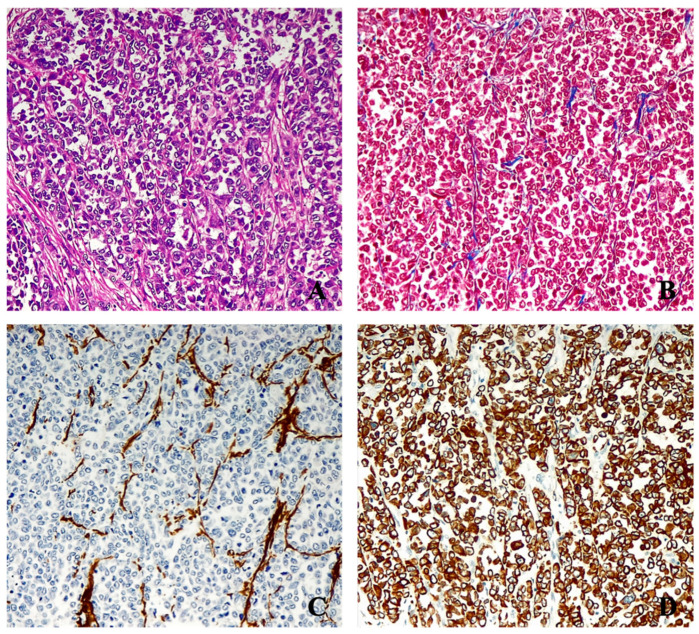

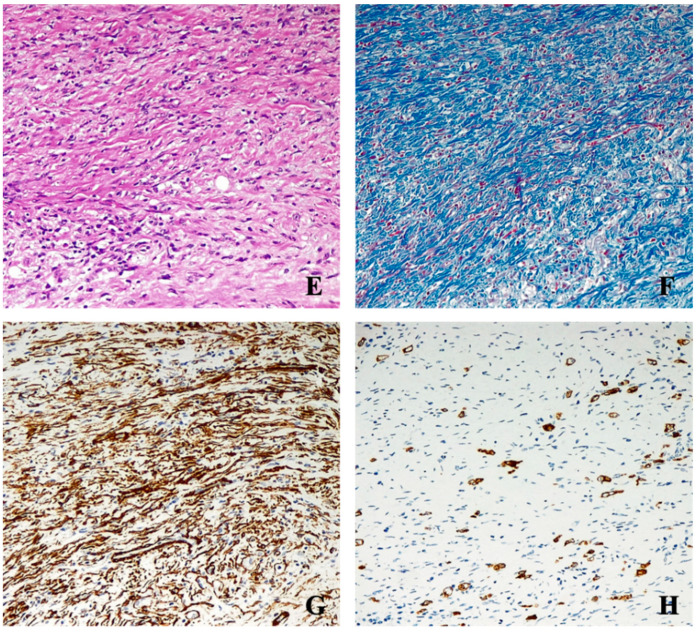
Representative histopathological features of stroma-poor (**upper panel**) and stoma-rich (**lower panel**) G. Following hematoxylineosin staining (**A**,**E**); Masson’s trichrome staining highlighting the large amount of neoplastic cells with a scant interposed stroma in the case of the SP group (**B**) and abundant stromal collagen fibers with only sparse neoplastic cells in the case of the SR group (**F**). Staining for α-smooth muscle actin further highlights the relatively scarce presence of CAFs in the SP group (**C**) in contrast to the numerous ones detected in the SR group (**G**). Staining for cytokeratin 8/18 reveals the large amount of neoplastic epithelial cells constituting the lesion in the SP cohort (**D**) while only scattered neoplastic cells can be seen in the SR cohort (**H**).

From formalin-fixed paraffin-embedded tissues specimens, two representative samples for each case were chosen and the representative regions of the tumor lesion were delineated for targeted tissue extraction and Tissue Microarray (TMA) construction (Figure 1).

To better assess the intratumoral heterogeneity, 3 cores of 1 mm in diameter were selected from each of the donor blocks, for a total of 6 cores for each patient sample. Then, a total of 450 cores of neoplastic tissue, alongside 20 cores of healthy gastric wall tissue (used as internal controls in each TMA), were transferred to 5 recipient paraffin blocks which were assembled using the tissue microarrayer Galileo TMA CK3600 Computer Driven (Integrated Systems Engineering S.r.l., Padova, Italy) and each TMA was sectioned at a thickness of 3 µm. The sections were stained with hematoxylin and eosin for morphological evaluation and with Masson’s trichrome stain and immunohistochemical stains to assess the proportion of epithelial neoplastic component and stromal amount. Immunohistochemistry was performed using antibodies against cytokeratin 8/18 (clone 5D3, Novocastra, Shanghai, China) and α-smooth muscle actin (α-SMA; clone 1A4, Dako, Glostrup, Denmark). The staining was carried out in an automated stainer, Leica Bond-III (Leica Biosystems, Wetzlar, Germany), using the ‘Bond Polymer Refine Detection’ system, according to manufacturer’s protocol. The stained slides were independently reviewed by two surgical pathologists (A.T., C.C.) to assess the intra-tumoral proportion of neoplastic cells, as highlighted by the epithelial marker cytokeratin 8/18, and the intra-tumoral stroma content, defined by the α-SMA positivity associated with cancer-associated fibroblasts (CAFs) deemed to correspond to the primary cellular component of the intra-lesional stroma. On the basis of the obtained staining patterns, the cases were divided in “stroma-rich” and “stroma-poor” groups, as previously described [17].

In the stroma-poor neoplasms, more than 50% of the tumor mass was composed of neoplastic cells and the rest of the tumor consisted of interposed stroma. On the contrary, in the stroma-rich neoplasms, the stroma represented more than 50% of the tumor mass and the cancer cells constituted a minor part.

### 2.3. Gene Expression Analysis

We performed a computational transcriptional analysis using the cBioPortal software and RNA-seq data from 82 GC PCs, which were divided into 69 diffuse-type GC cases and 13 signet ring cell GC cases, all part of the Firehose Legacy GC series of TCGA (18). The KRT8 mRNA expression levels were used to classify PCGC into SP (stroma poor) (KRT8-low group) and SR (stroma rich, KRT8-high group). Transcriptional signatures associated with the two PCGC KRT8-high and KRT8-low groups were established through the cBioPortal software [18]. The top-ranked 100 genes of each subgroup were considered for subsequent evaluation of the data sets.

The cellular localization of the proteins encoded by the top-ranked 100 genes preferentially expressed in the KRT8-high or KRT8-low groups and their abundance in the subcellular compartments were defined using the SubcellulaRVis software (enrichment visualization of protein localization software [19].

The determination of the gene expression pattern related to cell signaling pathways localized in the extracellular compartment was performed using the Enrichr software [20].

The overall survival (OS) of 240 diffuse GC patients was calculated through the KM plotter software [21]. The parameters used to calculate OS are the Lauren classification diffuse, auto selecting the best cutoff, splitting patients by median, compute median survival, censored at threshold, user selected probe set, and exclude biased arrays.

### 2.4. Statistical Methods

The levels of statistical significance of the differences observed between stroma-poor and stroma-rich cases were evaluated by Fisher’s exact test for nominal variables and by Wilcoxon-Mann-Whitney rank-sum test for ordinal variables (pT, pN, curativity) and quantitative variables with skewed distribution (age, number of excised or positive lymph nodes). Three deceased patients, whose dates of death were not available, were assumed to have died at the end of the follow-up period. The proportional-hazards assumption of the Cox model was tested on the basis of Schoenfeld residuals. In addition, the proportionality assumption was checked by graphic methods; it was verified whether the ln[-ln[survival]] curves for each category of risk factors were parallel, when plotted vs. ln[analysis time].

## 3. Results

### 3.1. Demographic, Histopathological and Clinical Characteristics at Baseline

According to the stromal proportion (50% cut-off within the tumour mass), the case series was divided into stroma-poor (48 cases) and stroma-rich (27 cases).

Female patients represented two thirds of the stroma-rich cases, and less than half of the stroma-poor ones (*p* = 0.150). The “pure” Signet Ring Cell histotype accounted for about 20% of the stromapoor cases but represented only one of the stroma-rich cases (*p* = 0.083).

Stromal status was observed to be significantly associated with the depth of tumor invasion (*p* = 0.002): pT1/pT2 tiers were not observed among stroma-rich cases, while representing 31.2% of the stroma-poor cases; on the other hand, nearly all (88.9%) stroma-rich cases could be classified as pT4. In addition, both nodal and distant metastases were more frequent among stroma-rich cases (*p* = 0.074 and *p* = 0.030, respectively). Accordingly, curative interventions were less frequently achieved in stroma-rich cases than in stroma-poor cases (*p* = 0.055; Table 1)

### 3.2. Long-Term Prognosis

**Figure 2:**
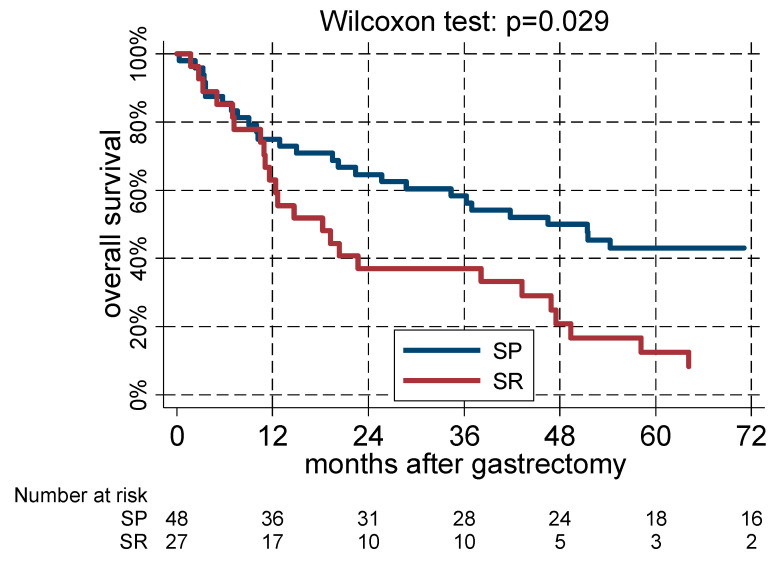
Evaluation of the 3 years survival in stroma-rich (SR) versus stroma-poor (SP) cases.

During the follow-up period, which comprised 57.28 person-years at risk, 52 deaths were recorded. The end of follow-up was considered 31/12/2017. Patients alive at follow-up (n = 23) had a median survival of 124 months (p25–p75: 71–140 months). The proportion of deceased patients was significantly higher in the stroma-rich (24/27 = 88.9%) than in the stroma-poor group (28/48 = 58.3%) (*p* = 0.008). Accordingly, 58% of the patients attested a three-year survival (at 95% Confidence Interval, 43–71%) in stroma-poor cancers versus 37% (20–55%) in stroma-rich cancers (*p* = 0.029). Of note, the two survival curves were superimposed until the 10th month of follow-up and started to diverge thereafter (Figure 2).

Stromal abundance remained a negative prognostic factor, also when controlling for sex and age distribution in a Cox regression model (Hazard Ratio of stroma rich with respect to stroma poor cancer = 2.54, 95% CI 1.43–4.48; *p* = 0.001) or sex, age, and SRC proportion (HR = 2.85, 95% CI 1.58–5.14; *p* < 0.001). The impact of the stromal content, although still significant, was slightly attenuated when considering pN status (HR = 2.02, 95% CI 1.11–3.67; *p* = 0.022). The impact of the stromal content lost statistical significance when evaluating also the relative depth of the tumor invasions (HR = 1.61, 95% CI 0.86–3.02; *p* = 0.134; Table 2). Sex and pN were the only significant predictors in the final model.

### 3.3. Enrichment in Cell Content and Predicted Function of Genes Upregulated in the KRT8-High versus KRT8-Low PCGC Groups

We subdivided 82 PCGC specimens into two groups of 41 samples each and these groups into KRT8-high (higher than the median KRT8 transcript levels) and KRT8-low (lower than the median KRT8 levels) and determined the listing of genes upregulated in the two subgroups using the cBioPortal software tool. Subsequently, we assigned the predicted subcellular localization of the proteins encoded by top-ranked 100 upregulated genes in the two groups and defined the enrichment of the proteins in the different subcellular compartments through the use of the SubcellulaRVis software tool (Table 3 and Table 4). In the specimens of the KRT8-high group, a significant gene enrichment was observed in extracellular space (FDR 0.00027), cytoplasm (FDR 0.0004), cytoskeleton (FDR 0.0018) plasma membrane (FDR 0.0020), and intracellular vesicles (FDR 0.0032) (Table 3). Proteins destined to the extracellular space were candidates for determining the characteristics of the stromal compartment of the 41 KRT8-high PCGC cases and the genes belonging to this category were ANXA2, FUCA2, JUP, LAMB3, LGALS3BP, OAS1, P4HB, PSMA5, RNPEP, S100A6, SPINT1, SPINT2, CDCP1, ANXA2P2, CLIC1, SFN, MOV10, ST14, PDIA4, MYDGF, LSR, AAMP, CEACAM1, GSS, ITGB4, KRT18, KRT19, PLS1, PTBP1, GPRC5A, GIPC1, CD2AP, PLXNB2, SDCBP2, EPS8L1, TMBIM1, EPS8L2, COASY, VPS25, CRB3.

In KRT8-low samples, the products of the top-ranked upregulated genes were found to be significantly enriched in the nucleus (FDR 0.014) and cytoskeleton (FDR 0.029; Table 4). Proteins with a putative distribution in the extracellular space included: EPHA3, F8, FGF7, SPARCL1, LGI2, IGIP, STX2, JAM3, ADGRB3, PKNOX2, PKD1, PYGM, andNIBAN1, whereas no significant accumulation was detected for the extracellular space (Table 4).

When we next performed a gene enrichment analysis for components of signal transduction pathways by selecting the genes encoding proteins with a predicted location in the extracellular space, we found 41 proteins of the upregulated KRT8-high group (Table 3) and 13 proteins upregulated in the KRT8-low group (Table 4). For the KRT8-high grouptop, a significant accumulation in the extracellular space was found for TCF4 (perturbation followed by expression) 6.4 *×* 10^−25^, MST1R (coexpression of ARCHS4 TF) 2.1 *×* 10^−24^, ZEB1 (ENCODE consensus TF, and ChEA from ChIP- X) 6.5 *×* 10^−5^, link with the Cadherin (GO Molecular Function 2023) 4.8 *×* 10^−8^, JUND MCF-7 HG19 (Encode TF Chip-Seq 2015) 5.3 *×* 10^−7^. In the KRT8-low group, a significant enrichment was highlighted for PMC8567138-Table 2.xlsx-Sheet1-IGFBP5 AND IGFBP7 NOT IGFBP3 NR4A2 (Rummagene transcription factors) 1.08 *×* 10^−8^, KLF9 (ARCHS4 TFs Coexp) 8.4 *×* 10^−7^, WT1 KO MOUSE GSE15325 CREEDSID GENE 2156 UP TF (Perturbations followed by expression) 7.1 *×* 10^−5^.

### 3.4. Enhanced Transcription of Genes Encoding Extracellular Proteins Associated with Increased Overall Survival in the KRT8-High Group and Reduced Overall Survival in the KRT8-Low Group of PC GC

**Figure 3:**
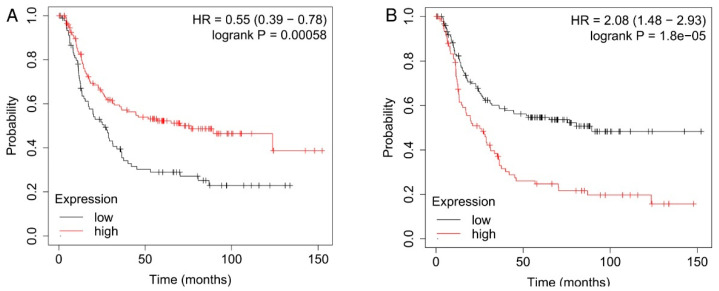
OS curves derived from expression levels of upregulated genes and having a preferential localization in the extracellular space lesions of the KRT8-high (**A**) or the KRT8-low group (**B**). The best cut-off of the average mRNA level of the 41 genes for the KRT8-high group and 13 genes for KRT8-low group was applied.

We analyzed the correlation between the transcriptional levels and overall survival (OS) in 240 diffuse GC cases with the best self-selected cut-off applied on the average expression levels of genes encoding extracellular proteins found to be upregulated in either the KRT8-high or the KRT8-low group. Higher mean levels of 41 upregulated genes in the KRT8-high group associated with augmented OS in patients with diffuse GC (*p* = 5.8 *×* 10^−4^) (Figure 3A). Genes found to most strongly correlate with OS were SPINT2 *p* = 2.1 *×* 10^−5^, DSI *p* = 5.4 *×* 10^−5^, and CEACAM1 *p* = 6.6 *×* 10^−5^ In contrast to the KRT8-high group, a higher expression level of 13 extracellular proteins in the KRT8-low group associated with poorer OS (*p* = 1.8 *×* 10^−5^; Figure 3B). In this case, genes with the stronger correlation were F8 *p* = 5.7 *×* 10^−8^, SPARCL1 *p* = 8.1 *×* 10^−6^, and JAM3 *p* = 2.0 *×* 10^−5^.

## 4. Discussion

Despite improvements in treatment strategies, the long-term survival of patients with GC remains unsatisfactory [1]. In recent years, great efforts have been undertaken to study the biology of this tumor type in greater depth to find potentially targetable molecular pathways of the neoplastic progression. Attention has been paid to the comprehension of the molecular interactions between cancer cells and tumor microenvironments as this is believed to be crucial in the promotion of tumor invasiveness, chemo-resistance, and metastatic spread [5,6,7].

In the present study, we set out to better understand the prognostic value of the stromal component in PCGC, uniquely focusing on this specific histological subtype defined as a distinct cancer entity by the WHO classification because of the increasing trend of poorly cohesive tumors in the Western world while GC is decreasing globally [22].

As in previous investigations [11,12,13,14,15], we subdivided our case series in two categories, “stroma-rich” and “stroma-poor” cases, by adopting the value of 50% of intralesional stromal content as a cut-off. We found out that the percentage of stromal content was significantly associated with the depth of the tumor invasion (*p* = 0.002): in the stroma-rich group there were no pT1 and pT2 cases but, of note, 88.9% of patients presented pT4 tumors. Accordingly, in the stroma-poor group, the 3-year survival was significantly higher when compared to the stroma-rich group (58% versus 37%; *p* = 0.029).

These findings are in line with what has been shown in another study [23] in which the 5-years OS was found to be 81% in the stroma-poor patients compared with 26% in the stroma-rich patients. Similarly, the 5-years disease-free survival rate was 88% in the stroma-poor patients compared with 26% in the stroma-rich patients (*p* < 0.001). Moreover, the relative stromal content of the lesions was found to constitute an independent prognostic factor. In the above-mentioned study, however, the evaluation of the stroma/tumor ratio was performed by simple morphological analyses of histological tumor sections. For this reason, in order to assess the proportion of epithelial neoplastic versus stromal component, we combined the hematoxylin-eosin and Masson’s trichrome stains for morphological assessment and immunohistochemistry for tumor and stromal markers cytokeratin 8/18 and α-smooth muscle actin (α-SMA).

On the other hand, discrepancies in the prognostic impact of the tumor/stroma ratio between our and other series of cancer cases [23] could be due to the absence of distinct subgroups analyses according to the tumor histotype (tubular vs. poorly cohesive) and this could be a confounding factor when assessing survival rates of the patients, given the well-known distinct biological behaviour of the two GC sub-types.

Indeed, low stromal content was identified as a significant independent risk factor for overall survival only in patients with intestinal-type GC [24]. Conversely, another study [25] has demonstrated that a high proportion of stroma is an independent prognostic factor in both intestinal and diffuse histological subtypes. These discrepancies may be due to the different pathological characteristics of the analysed patient populations, as well as to the different reference values used to define the stroma-rich versus stroma-poor groups. In particular, in one study [24], the median value of the stroma-tumor ratio was adopted, whereas in the other [25] the cut-off of 50% was used to discriminate between cases with poor and rich stromal components.

These earlier studies also failed to take into account that heterogeneous subtypes of cancer-associated fibroblasts (CAFs) can coexist and differently influence tumor cells in terms of invasiveness and metastatic spread, as has been demonstrated for pancreatic cancer [26].

We found here that an abundance of stroma was a significant risk factor for enhanced mortality rates by univariate regression analyses, whereas this parameter lost its significance when the patient samples were stratified according to the depth of the tumor invasion in multivariate analysis. Our results also suggest that enhanced stromal content is associated with a more advanced T stage. The absence of early lesions (pT1 and pT2) in the stroma-rich group may confirm that a close bidirectional relationship between tumor cells and CAFs may be responsible for reinforced tumor invasiveness in PCGC. It could be hypothesized that once a poorly cohesive tumor activates CAFs, these amplify and further stimulate tumor growth.

Of note, a Korean report [27] described the analysis of a series of signet ring cell (SRC) tumors and pointed out that a high stromal content is associated with the pathological stage of the tumor, as well as providing an independent negative prognostic impact in patients with pT3 and pT4 SRC tumors, but not in those with T1b and T2 cancers.

Our findings are consistent with the observations made in the Korean study with regard to the association between stromal abundance and the depth of tumor invasion in the PC gastric cancer subtype, but we could not find that this condition could constitute an independent prognostic factor. A possible explanation for this difference could be the histological heterogeneity of the cancers considered in the two previous studies. Indeed, Lee et.al., included “SRC” tumors, but no definition of the pathological classification that had been used (Japanese vs. WHO) to select the patients was made available. By contrast, for this study, we selected poorly cohesive tumours according to the WHO definition [28]. However, one of the main limitations of the present study is the evaluation of the intermediate cases that are difficult to classify. To cope with this issue, we provided an independent evaluation of each case by two expert pathologists with a high level of agreement observed.

Another important aspect is that if we limit our analysis to the pure SRC cases, which accounted for about 20% of the samples, we could observe they were not homogeneously distributed among the stroma-poor and stroma-rich groups: almost all of them fell into the stroma-poor group and only one case belonged to the stroma-rich group. A similar distribution was observed at mRNA level in the TCGA series. As such, a different selection of cases in the Korean report when compared to our present study may explain the different role assigned to the stromal content on tumor prognosis.

Interactions between cell surface proteins and different classes of ECM proteins are vital in many cellular processes, immune responses, in coordinating cellular differentiation, and in shaping cellular plasticity both in normal and tumor tissues [29,30]. In GC tissues, the ECM changes the onset and evolution of tumor cells [31]. Differentially distributed extracellular proteins between the KRT8-high and KRT8-low groups have not yet been studied in PC GC or other GC subtypes. According to stomach-related single-cell data reported in The Human Protein Atlas database, all extracellular proteins upregulated in the KRT8-low group are expressed in macrophages, endothelial cells, and fibroblasts. In contrast, the extracellular proteins upregulated in the KRT8-high group originate from stroma cells and/or specialized cells of the gastric mucosa (glandular cells). Extracellular proteins that originate from sources other than glandular cells are ANXA2, FUCA2, PSMA5, S100A6, MOV10, PTBP1, while FUCA2, CLIC1, PDIA4, MYDGF, AAMP, GPRC5A, GIPC1, TMBIM1 are expressed in both normal gastric stromal cells and glandular cells. Of course, extracellular proteins could also be ectopically expressed or lost by gastric cancer cells and contribute to cancer survival and evolution. Furthermore, the characteristics of the ECM and specifically of the protein matrix surrounding tumor cells are crucial factors in allowing the motility and dissemination capacity of tumor cells.

## 5. Conclusions

In conclusion, stroma-rich cancer represents an aggressive phenotype in gastric cancer, being associated with a more advanced depth of tumor invasion. Even though the relative abundance of intra-lesional stroma may not increase the prognostic information provided by TNM in gastric cancer, the expansion and enrichment of the stromal compartment, as a pathway of progression, could represent a potential target in the poorly cohesive gastric cancer subtype. Further studies are needed to demonstrate the role of the stroma as a determinant of tumor progression in this specific setting. The main limitation of the present study is its retrospective nature. The main strength is the clear selection of patients on a histopathological basis: to our knowledge this is the first report from the Western world focused on the role of stroma in the poorly-cohesive GC subtype.

## Figures and Tables

**Table 1:** Baseline demographic, clinical and histopathologic characteristics of patients presenting with stroma-poor or stroma-rich PC gastric cancer.

	Stroma-Poor (n = 48)	Stroma-Rich (n = 27)	*p* Value
Sex (men)	27 (56.3%)	10 (37%)	0.150
Age (median, p25–p75)	65.8 (50–74)	59.1 (49–70)	0.269
Site			0.725
Fundus	3 (75.0%)	1 (25.0%)	
Body	11 (55.0%)	9 (45%)	
Antrum	27 (65.9%)	14 (34.2%)	
Whole stomach	3 (50.0%)	3 (50.0%)	
Stump	2 (100%)	-----	
Signet Ring Cell (%)			0.083
≤90%	37 (80.4%)	25 (96.2%)	
>90%	9 (19.6%)	1 (3.9%)	
Depth of tumor invasion			**0.002**
pT1	14 (29.1%)	-----	
pT2	1 (2.1%)	-----	
pT3	6 (12.5%)	3 (11.1%)	
pT4	27 (56.3%)	24 (88.9%)	
Nodal metastases			0.074
pN0	19 (39.6%)	5 (18.5%)	
pN+	29 (60.4%)	22 (81.5%)	
pM+	3 (6.3%)	7 (25.9%)	**0.030**
Excised nodes (median, p25–p75)	36 (21–48)	33 (25–44)	0.873
Positive nodes (median, p25–p75)	2 (0–16)	9 (3–15)	0.232
Gastrectomy			0.803
Subtotal	32 (66.7%)	17 (62.9%)	
Total	16 (33.3%)	10 (37.0%)	
Lymphadenectomy			0.115
D1	6 (12.5%)	6 (22.2%)	
D2	28 (58.3%)	9 (33.3%)	
D3	14 (29.2.%)	12 (44.4%)	
Curativity			0.055
R0	43 (89.6%)	19 (70.4%)	
R1	5 (10.4%)	8 (29.6%)	
Adjuvant chemotherapy			1.000
Unknown or no	36 (75%)	20 (74.1%)	
yes	12 (25%)	7 (25.9%)	

Note: The significance was established by Fisher’s exact test for nominal variables, by Wilcoxon-Mann-Whitney rank-sum test for ordinal variables (pT, pN) and quantitative variables with skewed distribution (age, retrieved and positive lymph nodes).

**Table 2:** **The** hazard ratio (HR) of mortality from all causes and corresponding 95% confidence interval as a function of stromal content, controlling for sex, age, depth of tumor invasion, nodal metastases, and SRC proportion by a Cox regression model. The *p*-values were derived through the Wald test.

	HR (95% CI)	*p*-Value
**Sex: women vs. men**	**0.55 (0.31–0.99)**	**0.048**
Age (+10 years)	1.01 (0.89–1.34)	0.390
Depth of tumor invasion		
pT3 vs. pT1/pT2	1.71 (0.30–9.77)	0.548
pT4 vs. pT1/pT2	2.86 (0.91–9.05)	0.073
**Nodal metastases: pN+ vs. pN0**	**3.88 (1.58–9.54)**	**0.003**
SRC proportion: >90% vs. ≤90%	0.64 (0.27–1.52)	0.311
Stromal content: rich vs. poor	1.61 (0.86–3.02)	0.134

**Table 3:** Compartmental distribution of genes found to be upregulated in 41 KRT8-high PCGC cases.

Compartment	*p*-Value	FDR	n	Genes
Extracellular space	1.94 × 10^−5^	0.00027	41	ANXA2,FUCA2,JUP,LAMB3,LGALS3BP,OAS1,P4HB,PSMA5,RNPEP,S100A6,SPINT1,SPINT2,CDCP1,ANXA2P2,CLIC1,SFN,MOV10,ST14,PDIA4,MYDGF,LSR,AAMP,CEACAM1, GSS,ITGB4,KRT18,KRT19,PLS1,PTBP1,GPRC5A,GIPC1,CD2AP,PLXNB2,SDCBP2,EPS8L1,TMBIM1,EPS8L2,COASY,VPS25,CRB3
Cytoplasm	3.12 × 10^−5^	0.00040	77	AP1S1,PSEN1,WDR45B,VPS25,MOV10,ANXA2,ANXA2P2,BIRC5,BCL2L1,CLIC1,SFN,JUP,KRT8,KRT18,LLGL2,LMO7,OAS1,SLC22A18,PLS1,PTPRH,S100A6,PHLDA2,ARHGEF5,OASL,RPS6KA4,TRAF4,SPINT2,GIPC1,PKP3,TTLL12,CD2AP,POC1A,PLEK2,SDCBP2,SIRT7,DOK4,MYO19,PSRC1,TUBA1C,TRIM15,MARVELD3, DTX2, TEDC1, RNF149, FAM83H,BAK1,SLC25A10,HK2,MRPL44,COASY,RAB5IF,FUCA2,CYB561,TMBIM1,CLN6,P4HB,PDIA4,AGAT2,ERO1A,TMEM33,MYDGF,SGPP2,NCLN,AAMP,GSS,KRT19,PSMA5,TK1,TTLL4,EPS8L1,CNOT11,EPS8L2,ABHD17C,RHOV,CEACAM1,GPRC5A,LGA”S3BP
Cytoskeleton	0.00015	0.0018	24	POC1A,PSRC1,ARHGEF5,BCL2L1,HK2,PSEN1,TEDC1,KRT18,TTLL12,JUP,P4HB,TRAF4,PLEK2,TTLL4,TUBA1C,BIRC5,KRT19,PLS1,CD2AP,MYO19,AAMP,LLGL2,FAM83H
Plasma membrane	0.00018	0.0020	45	JUP,PKP3,AAMP,ANXA2,ANXA2P2,CEACAM1, CLIC1, GPR35,GPR39,HK2,ITGB4,KRT19,LLGL2,SLC22A18,PSEN1,RNPEP,S100A6,SPINT1,ST14,ARHGEF5,GPRC5A,TRAF4,AGAT2,SPINT2,CD2AP,PLEK2,CNNM4,SDCBP2,LSR,EPS8L1,ABHD17C,TMBIM1,EPS8L2,CDCP1,MRPL44,MFSD5,CRB3,RHOV,PTPRH,PLXNB2,AP1S1,KRT18,P4HB,KRT8,LMO7
Intracellular vescicles	0.00032	0.0032	24	VPS25,ANXA2,CLN6,PSEN1,GIPC1,ABHD17C,TMBIM1,RHOV,AP1S1,CD2AP,CEACAM1, GPRC5A, BCL2L1, LGALS3BP, MARVELD3, PSMA5, FUCA2, AGPAT2, JUP, ANXA2P2,P4HB,PDIA4,TMEM33,CYB561

**Table 4:** Compartmental distribution enrichment of genes upregulated in the KRT8-low PC GC group.

Compartment	*p*-Value	FDR	n	Genes
Nucleus	0.0010	0.014	55	PHF21A,FOXN3,ITPKB, MEF2C, NAP1L3, PKD1, POU6F1, MAPK10, STAT5B, TACC1, ZEB1, ZNF157, ZSCAN26, PRDM2, EPM2A, RGS9, TOX, AKT3, CAMTA2, SYNE1, KAT6B, CLIP3, CCDC69, DNAJC27, FAM120C,ZNF471,ZNF667,ZNF671,RNF146,THAP2,NRIP2,FAM172A,ZNF333,ZNF512,ZMAT1,ZNF542P,ZNF781,SYNPO2,ZNF25,ZNF546,NEXMIF,ZC3H6,ITPR1,DTNA,EPHA3,GRIK5,PPP3CB,GIT2,KLF12,KLF8,CEP68,PKNOX2,PWAR5,FILIP1,OSBPL6
Cytoskeleton	0.0023	0.029	21	KATNAL1, PGM5, SYNPO2, CEP68, TACC1, MAP7D3, SNAP25, TMOD1, FNBP1, SYNE1, TTLL7, CNR1, EPHA3, FILIP1, PKNOX2, DUSP22, JAM3, CLIP3, DTNA, CCDC69, NEXMIF
Extracellular region	0.99	0.99	13	EPHA3,F8,FGF7,SPARCL1,LGI2,IGIP,STX2,JAM3,ADGRB3, PKNOX2, PKD1, PYGM,NIBAN1

## Data Availability

Data represented or analyzed in this study are openly available as such or can be obtained by means of the software: Stomach Adenocarcinoma, Firehose Legacy Study at https://gdac.broadinstitute.org/runs/stddata__2016_01_28/data/STAD/20160128/ (accessed on 20 December 2023) and https://www.cbioportal.org/study/summary?id=stad_tcga (accessed on 20 December 2023); SubcellulaRVis software: Enrichment visualization of protein localization at http://phenome.manchester.ac.uk/subcellular/ (accessed on 20 December 2023); KM plotter at https://kmplot.com/analysis/index.php?p=service&cancer=pancancer_rnaseq (accessed on 20 December 2023); Enrichr at https://maayanlab.cloud/Enrichr/ (accessed on 20 December 2023). Data supporting this study are available from the Lead Contact upon reasonable request.
